# Downregulation of G2/mitotic-specific cyclinB1 triggers autophagy via AMPK-ULK1-dependent signal pathway in nasopharyngeal carcinoma cells

**DOI:** 10.1038/s41419-019-1369-8

**Published:** 2019-01-30

**Authors:** Xianhe Xie, Wanzun Lin, Weili Zheng, Ting Chen, Haitao Yang, Lijun Sun, Fei Huang, Zili Wang, Heng Lin, Long Chen, Jun Liu, Liyan Yang

**Affiliations:** 1 Department of Chemotherapy, The First Affiliated Hospital of Fujian Medical University, 20th Chazhong Road, 350005 Fuzhou, Fujian China; 2 Department of Central Laboratory, The First Affiliated Hospital of Fujian Medical University, 20th Chazhong Road, 350005 Fuzhou, Fujian China; 3 Department of Intensive Care Unit, The First Affiliated Hospital of Fujian Medical University, 20th Chazhong Road, 350005 Fuzhou, Fujian China; 4 Department of Nephrology, The First Affiliated Hospital of Fujian Medical University, 20th Chazhong Road, 350005 Fuzhou, Fujian China

## Abstract

CyclinB1 is a regulatory protein involved in mitosis. Multiple lines of evidence indicate that cyclinB1 depletion constrains proliferation and induces apoptosis in human tumor cells. The cells become susceptible to suffer a critical situation when cyclinB1 is downregulated. Autophagy is a major intracellular degradation system that recycles nutrients, removes damaged organelles, and promotes cell survival under stressful conditions, whereas the role of autophagy in cyclinB1-deprived neoplastic cell as well as the underlying molecular mechanism remains obscure. Here we pioneeringly elaborated that specific knockdown of cyclinB1 triggered autophagy via AMPK-ULK1-dependent signal pathway through the elevation of ROS, rather than ATP in the cell lines of CNE-1 and CNE-2. Moreover, ROS scavengers demonstrated that the observed effect of cyclinB1 silencing on AMPK phosphorylation was ROS dependent. Additionally, double knockdown of AMPK and cyclinB1 evidently abrogated cyclinB1 silencing-induced autophagy. Summarily, this study first revealed that downregulation of cyclinB1 induced autophagy via AMPK-ULK1-dependent signal pathway, which represents a key step toward unveiling the mechanism how cell cycle checkpoint proteins regulate autophagy.

## Introduction

The notion that autophagy is associated with either cell survival or cell death has been established by compelling functional researches undertaken over the past decades. Under conditions of severe stress, excessive autophagy induces cell death^1^. Alternatively, under some circumstances, moderate autophagy serves as part of normal metabolism to remove damaged proteins and organelles, which is imperative to sustain cell homeostasis^2,3^.

Dysregulation of the cell cycle checkpoint proteins, such as cyclinB1, cyclin D1, cyclin-dependent kinase 1 (CDK1), CDK4 and CDK6, is a key hallmark of cancer, generating uncontrolled cellular growth and tumorigenesis. Targeting cell cycle checkpoint proteins, such as palbociclib or ribociclib, a specific CDK4/6 inhibitor, has exhibited potent preclinical and clinical activities in numerous solid tumors^4^. It has been well documented that neoplastic cells activate autophagy in response to CDK4/6 inhibitors^5^, whereas little research has been conducted to probe the specific autophagy signal pathway mediated by cyclinB1 downregulation.

CyclinB1, a crucial cell cycle checkpoint protein, promotes mitosis and cyclinB1–Cdk1 involves the incipient events of mitosis, such as chromosome condensation, nuclear envelope breakdown, and spindle pole assembly. CyclinB1 depletion inhibits proliferation and triggers apoptosis in human tumor cells^6,7^, whereas the correlation between cyclinB1 depletion and autophagy remains to be ascertained.

To address this issue, we aimed to illuminate whether downregulation of cyclinB1 triggered autophagy as well as the underlying molecular mechanism. Double knockdown of AMPK and cyclinB1 was performed and cyclinB1 silencing-induced autophagy was evidently abrogated. Our results demonstrated that autophagy was induced by knockdown of cyclinB1 in nasopharyngeal carcinoma cell (CNE-1 and CNE-2 cells), which was mediated by activation of the AMPK-ULK1-dependent pathway.

## Results

### Specific downregulation of cyclinB1 induces autophagy in CNE-1 and CNE-2 cells

Double thymidine (TdR; 2.5 mmol/L) blocking could efficiently synchronize the cells to S phase. Then the cell viability was desirable and harvested for transfection experiments.

**Fig. 1:**
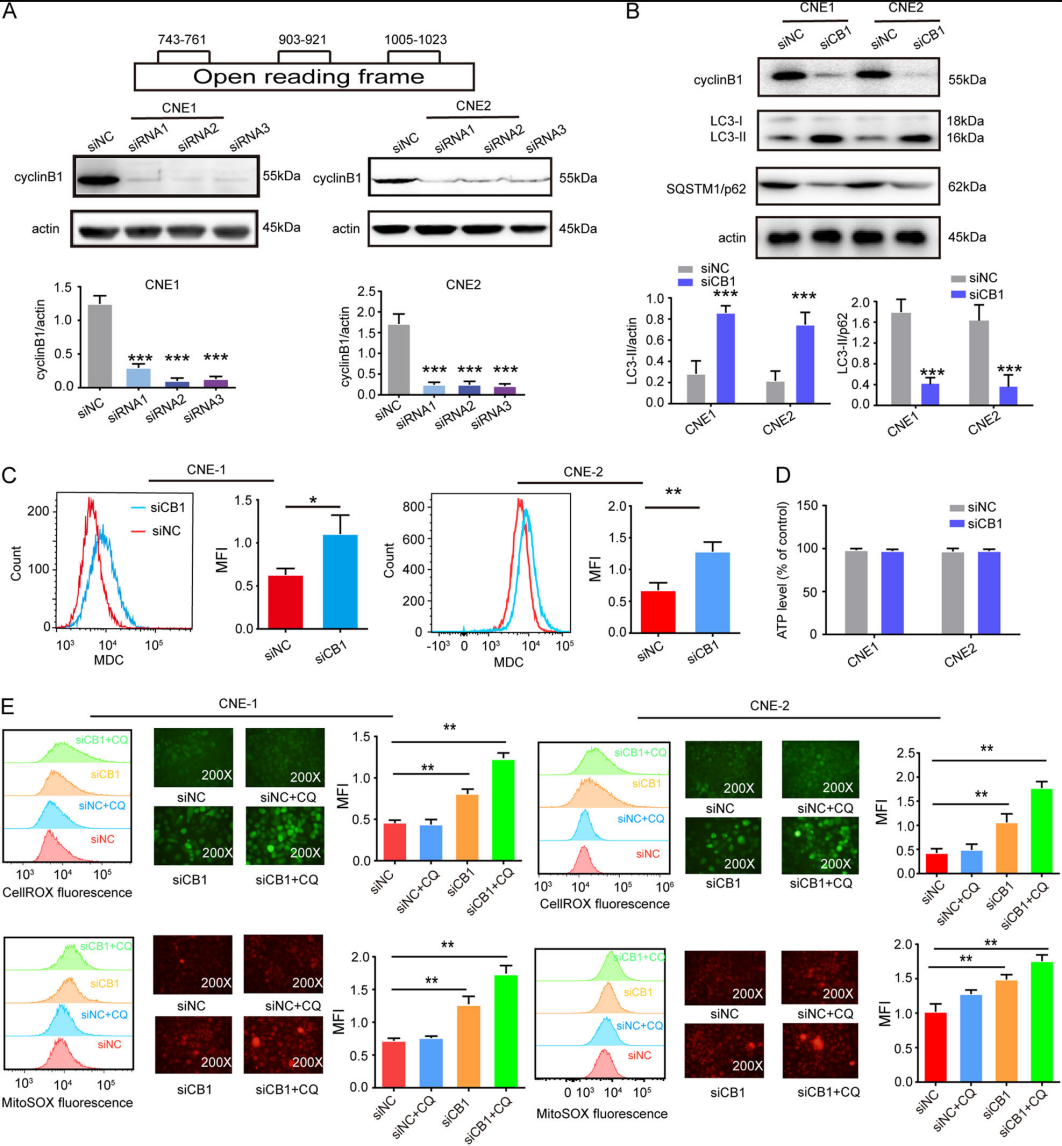
Downregulation of cyclinB1 induced reactive oxygen species (ROS)-mediated autophagy. **a** Three small interfering RNAs (siRNAs) were designed against the open reading frame of cyclinB1 mRNA, and silencing efficiency was detected by the western blot analysis. **b** Western blot for LC3B I, II, and p62 on treatment with non-coding siRNA (siNC) or cyclinB1 siRNA (siCB1) for 72 h. **c** Measurement of monodansylcadavarine-positive acidic vesicles, including autophagosomes, in CNE-1 and CNE-2 cells treated with siNC or siCB1 for 72 h by flow cytometry. Detection of **d** ATP and **e** cellular ROS and MitoSOX levels in both CNE-1 and CNE-2 cells upon transfection with siNC or siCB1 for 72 h. All data represented mean ± s.d. from three independent experiments; *P* values were calculated in comparison with cells treated with siNC (control) unless indicated. NS: *P* > 0.05; *P < 0.05; **P < 0.01; ***P < 0.001; ****P < 0.0001

Three small interfering RNAs (siRNAs) were designed against the open reading frame of cyclinB1 mRNA (Fig. 1a). Western blot showed that the protein level of cyclinB1 standardized to β-actin was apparently declined after transfected with each of the cyclinB1 siRNAs for 72 h in CNE-1 and CNE-2 cells (Fig. 1a).

It is hypothesized that autophagy in neoplastic cells was provoked in response to cyclinB1 depletion under stressful conditions. To validate this assumption, autophagy was assessed by: (i) monodansylcadavarine (MDC) staining, (ii) transmission electron microscopy (TEM), (iii) RFP-GFP-LC3B puncta, (iv) immunofluorescence, (v) transcriptome sequencing, and (vi) western blot analysis of LC3B-II (autophagosomal surface protein) and p62 (SQSTM1, an autophagic substrate).

**Fig. 2:**
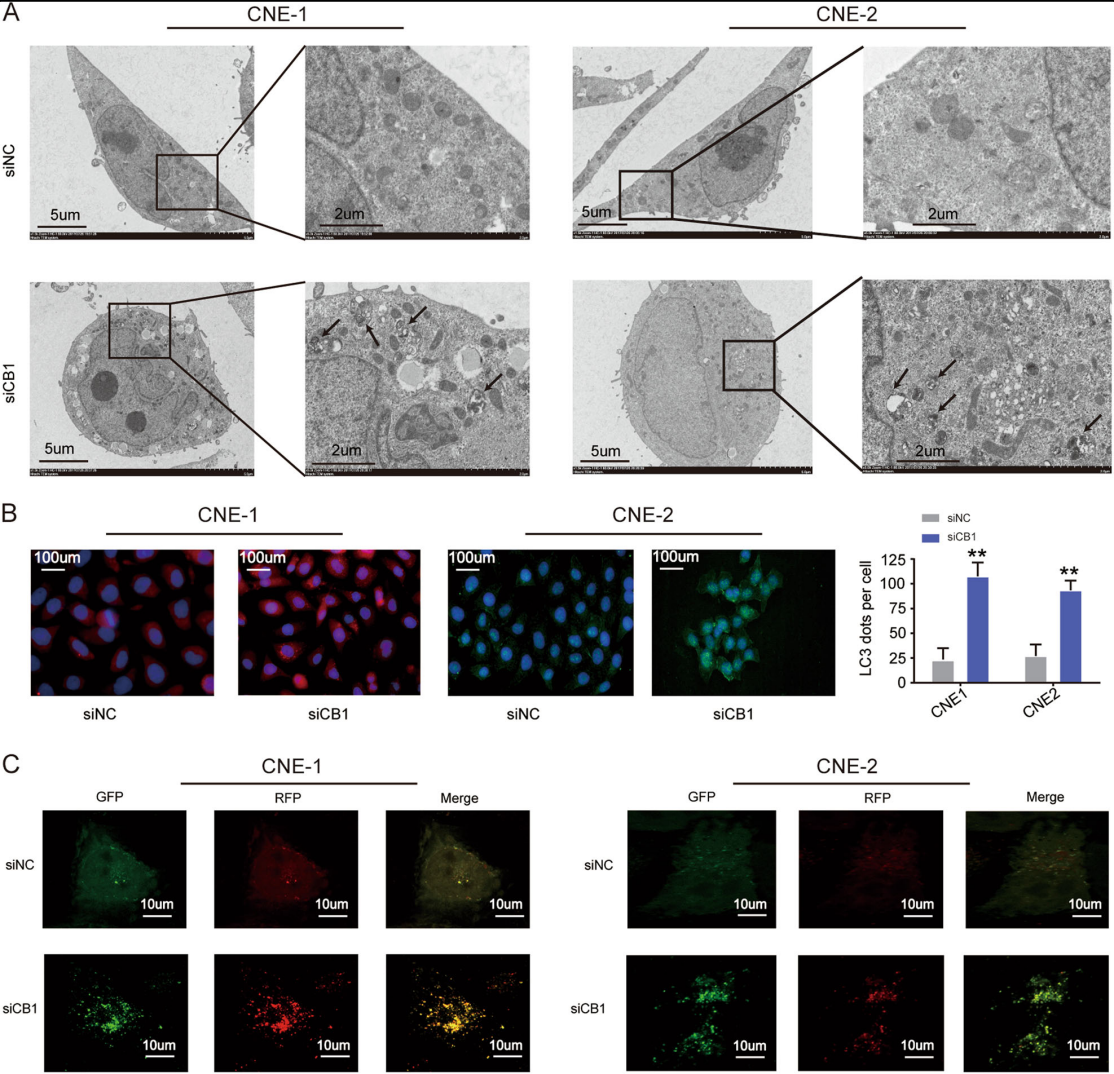
Detection of autophagosome. **a** Representative transmission electron microscopic microphotograph of cells treated with siNC or siCB1 for 72 h. Black arrows indicated double-membrane autophagosome. Scale bars, 5 µm and 2 µm. Quantification of endogenous LC3 dots by **b** immunofluorescence and **c** RFP-GFP-LC3B puncta by RFP-GFP-LC3B dual reporter assay. All data represented mean ± s.d. from three independent experiments; *P* values were calculated in comparison with cells treated with siNC (control) unless indicated. NS: *P* > 0.05; *P < 0.05; **P < 0.01; ***P < 0.001; ****P < 0.0001

The siRNA2 against cyclinB1 of CNE-1 and CNE-2 cells markedly elevated the amount of LC3B-II (Fig. 1b) and MDC (Fig. 1c) but attenuated p62 (Fig. 1b). Then TEM and LC3B immunofluorescence assays, which represented endogenous autophagosomes, displayed the noticeable accumulation of double-membrane electron-dense autophagosomes and LC3 puncta, respectively, in nasopharyngeal carcinoma cells treated with cyclinB1 siRNA2 (siCB1) (Fig. 2a, b).

Next, an intact autophagic flux following cyclinB1 siRNA treatment was identified by: (i) flux ratio (LC3B-II to actin and LC3B-II to p62) and (ii) RFP-GFP-LC3B dual-reporter assay. Cells treated with siCB1 exhibited higher flux ratios: the significant elevation of both LC3B-II to actin and LC3B-II to p62 (Fig. 1b) and the escalation in both RFP+ GFP+ puncta (autophagosomes) and RFP+ puncta (autophagolysosomes) (Fig. 2c). All evidenced an intact autophagic flux.

### CyclinB1 downregulation mediated autophagy via AMPK-ULK1-dependent pathway

To verify the specific autophagy signal pathway elicited by cyclinB1 depletion, we conducted transcriptome sequencing (RNA-Seq), quantitative polymerase chain reaction (q-PCR), and western blot analysis.

**Fig. 3:**
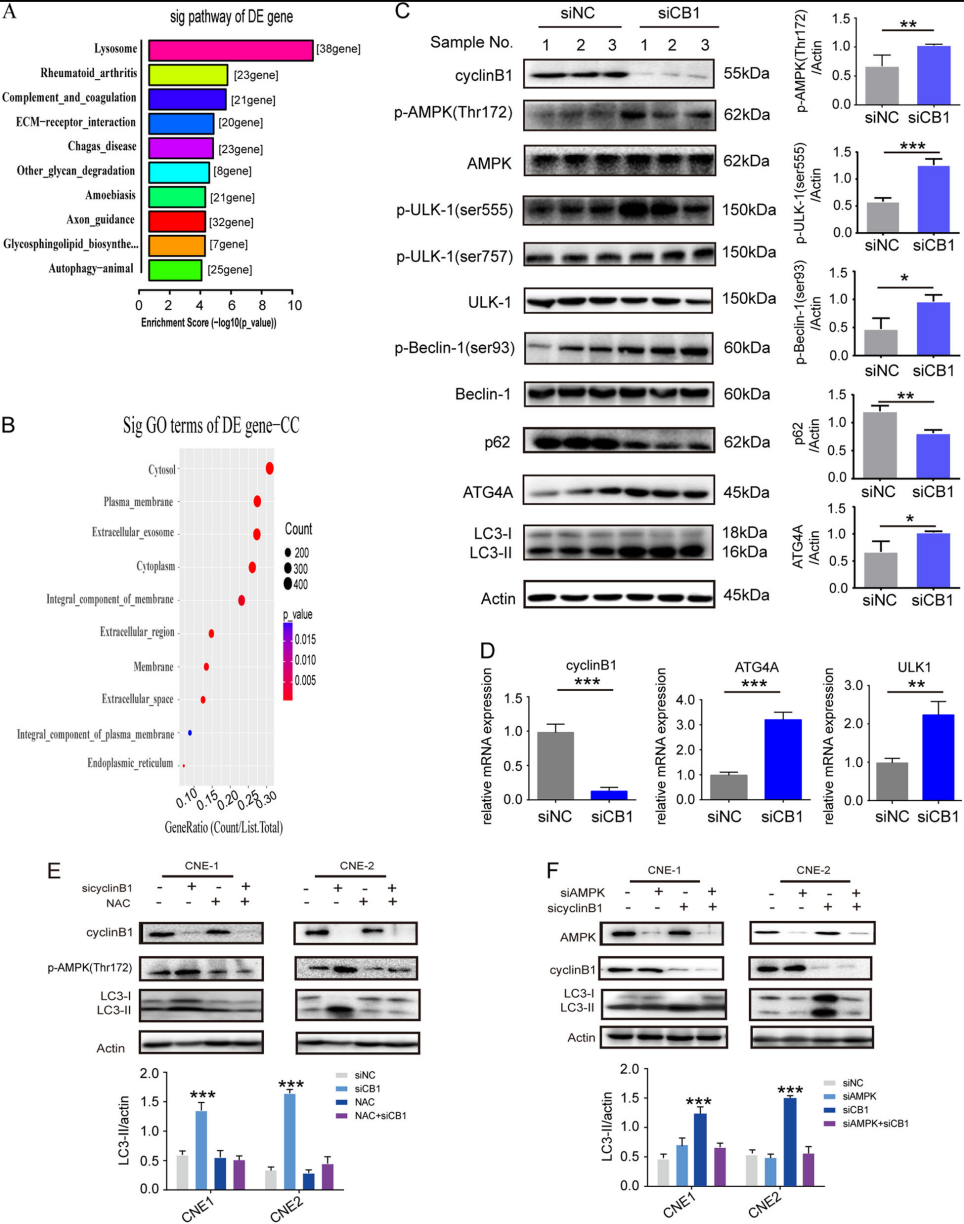
Downregulation of cyclinB1 triggered autophagy via AMPK-ULK1-dependent signal pathway. **a** The Kyoto Encyclopedia of Genes and Genomes pathway analysis of differentially expressed genes (DEGs) in siNC- and siCB1-treated cells. **b** Gene Ontology analysis of the genes related to cyclinB1 knockdown in CNE-1 and CNE-2 cells. **c** Western blot analysis of proteins related to autophagy signal pathway. **d** Real-time PCR analysis of proteins related to autophagy signal pathway. **e** *N*-acetylcysteine 10 mM remarkably attenuated AMPK phosphorylation and LC3-II level in cyclinB1 knockdown cells. **f** Double knockdown of AMPK and cyclinB1 evidently abrogated cyclinB1 silencing-induced autophagy. All data represented mean ± s.d. from three independent experiments; *P* values were calculated in comparison with cells treated with siNC (control) unless indicated. NS: *P* > 0.05; *P < 0.05; **P < 0.01; ***P < 0.001; ****P < 0.0001

A succession of autophagy-related genes (ATG) and various kinases are involved in the process of autophagy. We interrogated whether silencing of cyclinB1 contributed to the regulation of specific proteins of the ATG family. According to RNA-Seq, ULK1 (ATG1), ATG4A, ATG8A (GABARAP), and ATG16L2 were upregulated in the cyclinB1 siRNA treatment group (Fig. 3a, b). Next, q-PCR certified the upregulation of ULK1 and ATG4A, but not of ATG8A and ATG16L2 (Fig. 3d). Interestingly, although mRNA expression of ULK1 was upregulated, no difference in the protein level of ULK1 was observed between the cyclinB1 siRNA and control group based on western blot analysis (Fig. 3c). Moreover, ATG4A protein was increased in the cyclinB1 siRNA group. Accordingly, ULK1, AMPK, and beclin1 were activated via being phosphorylated at Ser555 (not at Ser757), Thr172, and Ser93, respectively. An evident decline of p62, an autophagic substrate, also occurred in the cyclinB1 siRNA group (Fig. 3c).

### AMPK is activated via the elevation of reactive oxygen species (ROS), rather than ATP

Previous evidence argued that AMPK was generally activated by an increment of AMP/ATP ratio^8,9^. In contrast, recent researches highlighted the essential role of ROS in AMPK activation^10^. Our data revealed that ATP levels were almost constant between siCB1 and control group, supporting that ATP was not involved in autophagy induced by silencing of cyclinB1 (Fig. 1d). Instead, cellular and mitochondrial ROS levels was evidently elevated. These led to an activation of AMPK, which permitted persistent firing of the downstream signal transduction (Fig. 1e).

Consequently, the results indicated that autophagy induced by silencing of cyclinB1 was attributed to activation of AMPK-ULK1 pathway through the elevation of ROS, rather than ATP.

### The ROS and AMPK are imperative for autophagy in response to cyclinB1 silencing

In order to further validate whether AMPK and ROS are vital to autophagy induced by cyclinB1 knockdown, the effects of AMPK siRNA and ROS scavengers (*N*-acetylcysteine (NAC)) on autophagy in cyclinB1-depleted cells (both CNE-1 and CNE-2 cells) were analyzed. NAC remarkably attenuated AMPK phosphorylation and LC3-II level in cyclinB1 knockdown cells, indicating that ROS was responsible for cyclinB1 knockdown-induced autophagy (Fig. 3e). Furthermore, double knockdown of AMPK and cyclinB1 evidently abrogated cyclinB1 silencing-induced autophagy, implying that AMPK was also involved in autophagy mediated by cyclinB1 silencing (Fig. 3f).

### CyclinB1 overexpression predicts a poor prognosis in neoplastic disease

**Fig. 4:**
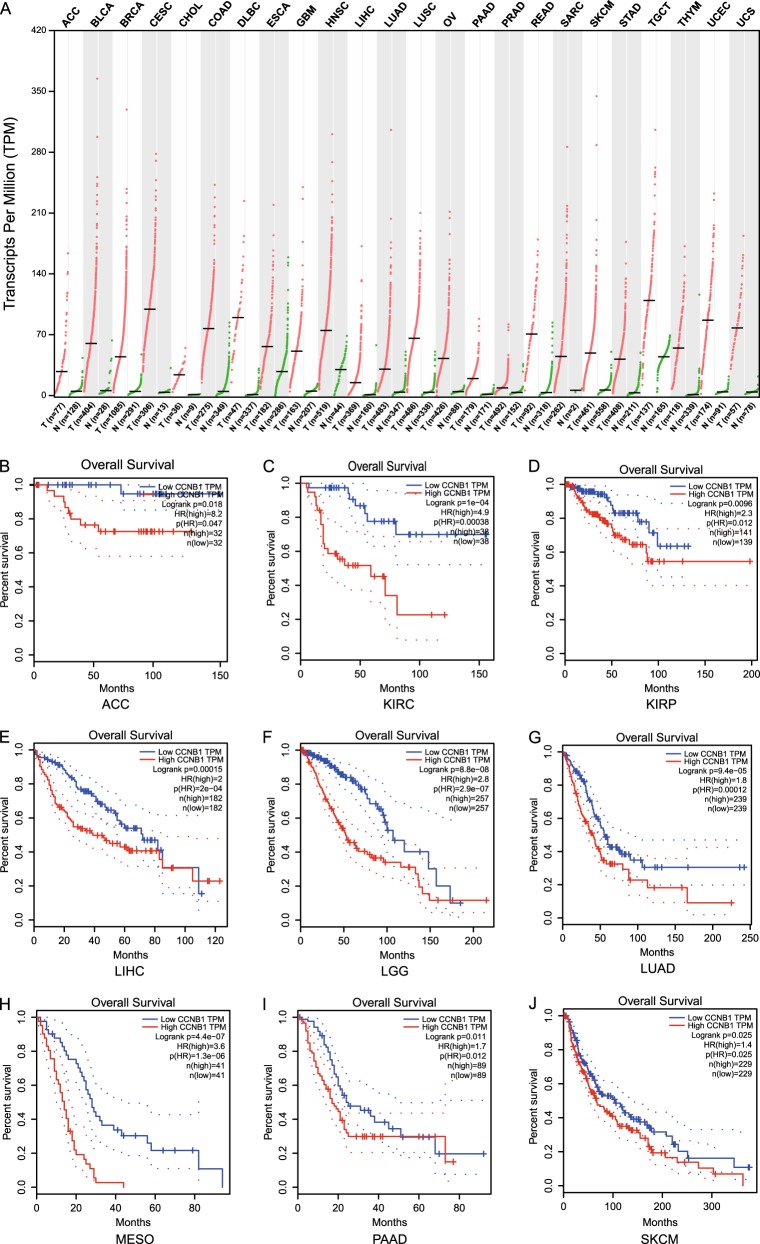
Correlation between cyclinB1 expression and prognosis in 31 types of tumors based on The Cancer Genome Atlas database **a** CyclinB1 was overexpressed in 31 types of tumors by analyzing the data from The Cancer Genome Atlas. **b**–**j** Patients harboring high expression of cyclinB1 possessed a poor prognosis in adrenocortical carcinoma, kidney renal clear cell carcinoma, kidney renal papillary cell carcinoma, liver hepatocellular carcinoma, brain lower-grade glioma, lung adenocarcinoma, mesothelioma, pancreatic adenocarcinoma, and skin cutaneous melanoma

The function of cyclinB1 is to drive cells from G2 to M phase but becomes dysregulated in neoplastic cells. Prior investigations exhibited that cyclinB1 was overexpressed in a variety of tumors and closely associated with the extent of tumor invasion and metastasis^11–14^. Here we performed a comprehensive analysis on 31 types of tumors from The Cancer Genome Atlas (TCGA) and GTEx projects applying GEPIA^15^ and found that 15 kinds of tumors overexpressed cyclinB1 (Fig. 4a). Furthermore, patients harboring high expression of cyclinB1 possessed a poor prognosis in adrenocortical carcinoma (ACC), kidney renal clear cell carcinoma (KIRC), kidney renal papillary cell carcinoma (KIRP), liver hepatocellular carcinoma (LIHC), brain lower-grade glioma (LGG), lung adenocarcinoma (LUAD), mesothelioma (MESO), pancreatic adenocarcinoma (PAAD), and skin cutaneous melanoma (SKCM) (Fig. 4b), denoting the dampening of cyclinB1 might serve as an attractive strategy for anticancer.

## Discussion

In this study, we first addressed that downregulation of cyclinB1 triggered autophagy via AMPK-ULK1-dependent signal pathway by the elevation of ROS.

**Fig. 5:**
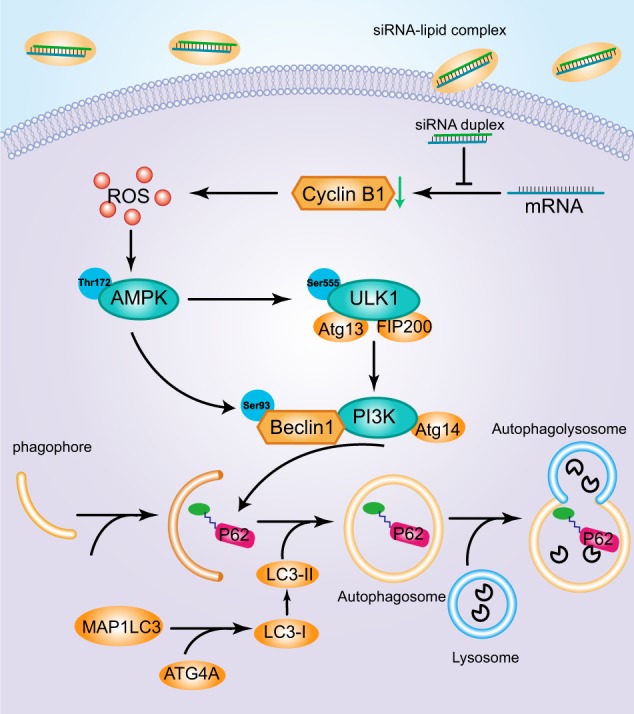
Signaling connections involved in autophagy pathways sensitive to cyclinB1 silencing in CNE-1 and CNE-2 cells. Silencing of cyclinB1 elevated the reactive oxygen species levels. This in turn directly activated AMPKα by phosphorylating at Thr172 and then activated ULK1 by phosphorylating at Ser555 leading to autophagy. Activated AMPKα also phosphorylated beclin-1 at Ser93 site, which further enhanced the activity of PI3K compound. Moreover, elevated ATG4a cleaved the C-terminal part of MAP1LC3 allowing the release of (LC3-I). Then a subpopulation of LC3-I was subsequently converted into a LC3-II. Additionally, p62 bound autophagosomal membrane protein LC3/Atg8, delivering p62-containing protein aggregated to the autophagosome, which led to a decline in p62 level. Arrows represented promotion events, blunt arrows indicated suppression events

Although the cellular conditions that trigger autophagy remain to be fully illuminated, diverse aberrations of nutrient deficiency, ROS, and DNA damage have been identified. These processes are coordinated by ATG proteins and various kinases, like AMPK and ULK1, functioned as crucial regulators in autophagy initiation and progression^16^. AMPK is an evolutionarily conserved serine/threonine kinase that plays a vital role in sustaining cellular metabolic balance^17^. A wealth of evidence confirms the critical role of AMPK in autophagy initiation. In the autophagy signal pathway, activated AMPK directly phosphorylates ULK1 at multiple sites, including Ser317, Ser555, Ser777^18,19^, and beclin-1 at Ser93 sites^20^. Conversely, ULK1, phosphorylated at Ser757, disrupts the interaction between ULK1 and AMPK^18^. It was historically deemed that AMPK was activated by an elevated AMP/ATP ratio due to cellular and environmental stress, but recent research advocated that ROS also exerted an evident impact on the activation of AMPK under certain conditions^9,21,22^. Our research exhibited that AMPK was activated via the elevation of ROS, rather than ATP, after cyclinB1 was diminished. The activated AMPK phosphorylated both ULK1 at Ser555 site and beclin1 at Ser93 site, ultimately potentiating the activity of the class III phosphatidylinositol 3-kinase complex. Moreover, p62 bound autophagosomal membrane protein LC3/Atg8, delivering p62-containing protein aggregated to the autophagosome, which in turn led to a decline in p62 levels during autophagy. Additionally, an elevated ATG4a level was observed in our research, which cleaved the C-terminal part of MAP1LC3 allowing the release of form I (LC3-I). Then a subpopulation of form I was subsequently converted to form II. Eventually, these kinases and ATG proteins were able to orchestrate multiple steps of autophagy progression and rationalize the complexities of autophagic program, as we portrayed in Fig. 5.

One of the hallmarks of cancer is the disorder of cell cycle. Prior surveys substantiated that cyclinB1 was abundant in various tumors and conferred on neoplastic cells traits associated with malignancy^11–14^. Here we intensively scrutinized the expression of cyclinB1 in 31 types of tumors by analyzing the data from TCGA and GTEx, which revealed that cyclinB1 overexpressed in vast majority of them, implying that cyclinB1 was closely associated with the biologic behavior of tumors. Of note, cyclinB1 overexpression predicted a poor prognosis.

In this study, the identification of specific autophagic signal pathway mediated by cyclinB1 depletion represents a key step toward unveiling the mechanism on how cell cycle checkpoint proteins regulate autophagy.

## Material and methods

### Reagents and antibodies

Antibodies against phospho-ULK (Ser757) (#6888, 1:2000), phospho-ULK1 (Ser555) (#5869, 1:2000), ULK1 (#8054, 1:2000), phospho-Beclin-1 (Ser93) (#14717, 1:2000), Beclin-1 (#3738, 1:2000), phospho-AMPKα (Thr172) (#2535, 1:2000), AMPKα (#2532 S, 1:2000), and p62/SQSTM1 (#5114, 1:2000) were obtained from Cell Signaling Technology (Cell Signaling Technology, Danvers, MA). Antibodies against cyclinB1 (ab32053, 1:3000), ATG4A (ab108322, 1:3000), LC3B (ab51520, 1:3000), and β-actin (ab8227, 1:3000) were ordered from Abcam (Abcam, Cambridge, UK). Goat-IgG Rabbit Polyclonal antibody (10285-1-AP, 1:5000) was purchased from Proteintech. ECL luminescence reagent (abs920) was obtained from Absin (Absin Bioscience Inc., Shanghai, China).

### Cell culture

CNE-1 and CNE-2 cells were gifted from Central Laboratory of The First Affiliated Hospital of Fujian Medical University and were cultured in RPMI-1640 (Invitrogen, Carlsbad, CA, USA) with 10% fetal bovine serum (HyClone, Logan, UT, USA).

### siRNAs and in vitro transfection

Three siRNA sequences targeting cyclinB1 were synthesized by Guangzhou Rui Bo Biological Technology. The siRNA1 targets at the sites 743–761 of the cyclinB1 open reading frame, siRNA2 at the sites 903–921, and siRNA3 at the sites 1005–1023. Cells were transfected with siRNAs using LipofectamineRNAiMAX transfection reagent (Invitrogen) based on the manufacturer’s instruction. Compactly, cells were seeded to a confluence of 50–60% and transfected with siRNAs (50 nM). Control cells were treated with NC-siRNA. The siRNA sequences were as follows.

CyclinB1 siRNA1: Sense, 5’-CCAAACCUUUGUAGUGAAUTT-3’,

CyclinB1 siRNA1: Antisense, 5’-AUUCACUACAAAGGUUUGGTT-3’;

CyclinB1 siRNA2: Sense, 5’-GGUUGUUGCAGGAGACCAUTT-3’,

CyclinB1 siRNA2: Antisense, 5’-AUGGUCUCCUGCAACAACCTT-3’;

CyclinB1 siRNA3: Sense, 5’-CCAUGUUUAUUGCAAGCAATT-3’,

CyclinB1 siRNA3: Antisense, 5’-UUGCUUGCAAUAAACAUGGTT-3’;

NC-siRNA: Sense, 5’-UUCUCCGAACGUGUCACGUTT-3’,

NC-siRNA: Antisense, 5’-ACGUGACACGUUCGGAGAATT-3’.

PRKAA1-siRNA: Sense, 5’-GAGGAGAGCUAUUUGAUUATT-3’,

PRKAA1-siRNA: Antisense, 5’-UAAUCAAAUAGCUCUCCUCTT-3’.

PRKAA2-siRNA: Sense, 5’-GCUGUUUGGUGUAGGUAAATT-3’,

PRKAA2-siRNA: Antisense, 5’-UUUACCUACACCAAACAGCTT-3’.

### Western blot analysis

Briefly, cells were lysed with RIPA buffer (150 mM NaCl, 10 mM Tris, pH 7.3, 0.1% sodium dodecyl sulfate (SDS), 1% Triton X-100, 1% deoxycholate and 5 mM ethylene-diaminetetraacetic acid) containing protease inhibitors. Lysates were then subjected to centrifugation at 15,000 r.p.m. for 15 min at 4 °C to obtain the protein lysates in the supernatant. Protein concentration was determined by Bradford Protein Assay dye (Bio-Rad), and 20 µg of protein per sample was analyzed on a 12% gel by SDS–polyacrylamide gel electrophoresis, transferred to a nitrocellulose membrane, blocked with 5% bovine serum albumin (BSA) in TBST buffer, incubated with primary antibodies at 4 °C overnight, washed 3 times for 15 min each in TBST at room temperature, incubated with horseradish peroxidase-conjugated secondary antibodies for 1 h at room temperature, and washed 3 times for 15 min each in TBST. The bands were then washed and developed using a FlourChemE system (Protein Simple) following the manufacturer’s instructions. The developed and scanned bands were then analyzed by the ImageJ software to obtain densitometric values.

### ROS measurement

Cellular and mitochondrial ROS were assessed by incubating cells with 2’,7’-dichlorofluorescin diacetate or MitoSOX Red mitochondrial superoxide indicator (ThermoFisher Scientific, Waltham, MA), respectively, followed by flow cytometric analysis. ROS levels were quantified as the mean florescence intensity (MFI). All flow cytometry was executed by BD Accuri C6 and analyzed with the FlowJo software.

### MDC measurement

After transfection for 72 h, cells were incubated with 50 mM MDC (Sigma-Aldrich) at 37 ℃ for 30 min, then collected, washed with phosphate-buffered saline (PBS), and suspended in a solution of PBS with 1% FBS. Samples were analyzed on the BD Accuri C6. Data were analyzed with the FlowJo software, and MDC levels were quantified as the MFI.

### Quantitative real-time PCR

Total mRNA was isolated from cells using Trizol (Invitrogen), and cDNA was synthesized from 100 ng of total RNA using the PrimeScript™ RT reagent Kit with gDNA Eraser (TaKaRa). q-PCR was performed with GoTaq qPCR Master Mix (Promega, Madison, USA) and Applied Biosystems 7500 Real-Time PCR Systems. All samples were normalized to β-actin mRNA levels.

### Intracellular ATP level measurement

The ATP levels in cells were detected using the Enhanced ATP Assay Kit (Beyotime Biotechnology) following the manufacturer’s instructions. Concisely, the assay buffer was gently mixed with the substrate at room temperature. The mixed reagent 100 µl was added into each well and incubated for 15 min at room temperature. Then luminescence was measured using a microplate reader (Beckman Coulter).

### Transmission electron microscopy

Cells were fixed in 2.5% glutaraldehyde and 0.1 M cacodylate buffer (pH 7.4) for 2 h following trypsinization and rinsed twice with precooled PBS. After washing with buffer solution, cells were post-fixed in 1% osmium tetroxide (OsO_4_) and 0.1 M cacodylate buffer (pH 7.4). Then the fixed cells were washed with PBS, dehydrated through different concentrations of ethanol, and embedded in epoxy resin. The ultrastructures of cells undergoing autophagy were observed and imaged under TEM (JEM-1200; Jeol Ltd, Tokyo, Japan) at 80 kV.

### Cell cycle synchronization

TdR double blocking was applied to synchronize cell cycle. Concisely, the cells with a confluence of 40–50% was incubated in a medium containing TdR (2.5 mmol/L) for 18 h (the first block), washed twice with PBS, changed into complete medium for 8 h, and then incubated for 20 h with a medium containing the same concentration of TdR (the second block), eventually with complete medium for 4 h.

### Cell immunofluorescence

The cells were seeded on slides and transfected with siRNAs. Twenty-four hours later, cells were washed 3 times with PBS, fixed with 4% paraformaldehyde for 10 min, washed another 3 times with PBS, permeabilized with 0.1% TritonX-100 for 10 min, and blocked with 5% BSA in PBS for 1 h. Subsequently, cells were stained with LC3B (1:200) for 1 h at room temperature, then with fluorescein isothiocyanate or Alex555 goat anti-rabbit antibody (1:100) for 1 h at room temperature, finally with 4,6-diamidino-2-phenylindole (1:10,000) for 5 min and photographed under a fluorescence microscope.

### Statistical analysis

Student’s *t* test, one-way analysis of variance, and log-rank test were used. *P* < 0.05 was considered statistically significant.
